# Characterisation of Bone Beneficial Components from Australian Wallaby Bone

**DOI:** 10.3390/medicines3030023

**Published:** 2016-08-26

**Authors:** Weiguo Lao, Xingliang Jin, Yi Tan, Linda Xiao, Matthew P. Padula, David P. Bishop, Brian Reedy, Madeleine Ong, Mohammad A. Kamal, Xianqin Qu

**Affiliations:** 1 School of Life Sciences, University of Technology Sydney, Ultimo 2007, NSW, Australia; weiguo.lao@uts.edu.au (W.L.); jinxingsz4@hotmail.com (X.J.); tanyiok@gmail.com (Y.T.); matthew.padula@uts.edu.au (M.P.P.); madeleine.l.ong@student.uts.edu.au (M.O.); prof.makamal@lycos.com (M.A.K.); 2 School of Mathematical and Physical Sciences, University of Technology Sydney, Ultimo 2007, NSW, Australia; linda.xiao@uts.edu.au (L.X.); david.bishop@uts.edu.au (D.P.B.); brian.reedy@uts.edu.au (B.R.); 3 Proteomics Core Facility, Faculty of Science, University of Technology Sydney, Ultimo 2007, NSW, Australia

**Keywords:** wallaby bone, mineral components, protein components, bone remodelling proteins, osteoporosis

## Abstract

**Background:** Osteoporosis is a condition in which the bones become brittle, increasing the risk of fractures. Complementary medicines have traditionally used animal bones for managing bone disorders, such as osteoporosis. This study aimed to discover new natural products for these types of conditions by determining mineral and protein content of bone extracts derived from the Australian wallaby. **Methods:** Inductively coupled plasma-mass spectrometry and Fourier transform infrared spectroscopic analysis were used for mineral tests, proteome analysis was using LC/MS/MS and the effects of wallaby bone extracts (WBE)s on calcium deposition and alkaline phosphatase activity were evaluated in osteogenic cells derived from adipose tissue-derived stem cells (ADSCs). **Results:** Concentrations of calcium and phosphorus were 26.21% and 14.72% in WBE respectively. Additionally, minerals found were wide in variety and high in concentration, while heavy metal concentrations of aluminium, iron, zinc and other elements were at safe levels for human consumption. Proteome analysis showed that extracts contained high amounts of bone remodelling proteins, such as osteomodulin, osteopontin and osteoglycin. Furthermore, in vitro evaluation of WBEs showed increased deposition of calcium in osteoblasts with enhanced alkaline phosphatase activity in differentiated adipose-derived stem cells. **Conclusion:** Our results demonstrate that wallaby bone extracts possess proteins and minerals beneficial for bone metabolism. WBEs may therefore be used for developing natural products for conditions such as osteoporosis and further investigation to understand biomolecular mechanism by which WBEs prevent osteoporosis is warranted.

## 1. Introduction

Osteoporosis is a medical condition in which the bones become brittle and fragile from a loss of tissue, typically as a result of hormonal changes or deficiency in calcium or vitamin D [1]. This increases the likelihood of complications such as fractures, which most commonly occur in the spine, wrist and hips, but can also affect the arm or pelvis. According to the International Osteoporosis Foundation (IOF), it is estimated that there are approximately 200 million osteoporosis patients worldwide, with a particularly high prevalence in females and the elderly [2]. In Australia, not only is there an aging population, but also relatively high rates of vitamin D deficiency. Moreover, osteoporosis heavily contributes to hospitalisation and related expenses costing approximately $1.9 billion per annum [3]. Preventative treatment of osteoporosis and related fractures for high risk groups is important. Therefore it is necessary to develop products suitable for long-term use [4,5,6]. A promising source of natural products for osteoporosis commonly uses plant-based agents such as marine plants and soybean products, which are rich in protein and calcium. Recently, the contents of animal bones have become of interest, with research into as many as 17 animals, including sika deer, red deer, pig, sheep, dog, and cow [7]. The bone extracts from these animals may contain active minerals and proteins such as Bone Morphogenetic Proteins (BMPs) that could positively impact bone and fat metabolism. Despite this, research at this stage is primarily observational with more investigation needed for the mechanisms of action of the active ingredients of these bone products.

The wallaby is an active, lean and wild animal unique to Australia, with over sixty different species. It is estimated that the four most common species of wallaby are numerically in excess of 50 million animals [8,9]. At present, the wallaby industry relies heavily on producing wallaby meat [10], while the bones are considered to be a waste product. For these reasons, we hypothesised that wallaby bone extracts may be a potentially efficacious treatment option for osteoporosis. In this study we aimed to identify and quantify the ingredients (mineral and protein) of wallaby bone extracts while also identifying the calcium to phosphorus ratio and heavy metal content. This was achieved by using inductively coupled plasma-mass spectrometry (ICP-MS) and Fourier transform infrared spectroscopy (FT-IR) spectroscopic analysis. Alongside this, liquid chromatography-mass spectrometry (LC-MS/MS) was used to perform proteomics analyses, which further clarified the wallaby bone protein composition and putative functions.

To our knowledge, this is the first scientific evaluation of Australian wallaby bone extracts using a Systems Biology approach, and may lead to the discovery of a potential alternative therapy for the treatment and prevention of osteoporosis.

## 2. Material and Methods

### 2.1. Animal and Bone Samples

Bennett’s wallaby *Macropus rufogriseus*, mixed genders, were harvested from Tasmanian farmlands in Australia. The fresh femur, tibia and fibula bones were collected and supplied by Lenah Game Meats. The bones of three individual wallabies were used in the physical testing and analysis processes. In brief, after harvesting, fresh bones were trimmed free of soft tissue and washed with cold phosphate buffer saline (PBS) to remove contaminants, wrapped with gauze pre-soaked in PBS containing a protease inhibitor cocktail, then stored at −20 °C overnight. Ten grams of the parietal bone fragments (about 10 mm × 1 mm size, 1 to 2 mm thick, *n* = 3) were frozen in liquid nitrogen and then ground up using a mortar and pestle for subsequent chemical analysis and protein determination [11].

### 2.2. Digestion Procedures for Wallaby Bone

Wallaby bone samples which contained fibula, femur and tibia bone specimens were dried overnight and weighed (about 0.150 g). The dried samples were then placed into a 15-mL Falcon tube (Fisher Scientific, Sydney, NSW, Australia). Optima grade nitric acid (0.8 M, 3 mL) and ultrapure hydrogen peroxide (30%, 1 mL) were then added to the tubes and capped. The tubes were placed on a hot plate at 45 °C for one hour. The temperature was then increased to 80 °C for 12 h. The final solution was quantitatively transferred to 50 mL plastic centrifuge tubes (Fisher Scientific, Sydney, NSW, Australia) and filled to the mark with ultrapure deionized water (>18 MΩ·cm^−1^) and weighed.

### 2.3. Inductively Coupled Plasma-Mass Spectrometry

The ICP-MS system (Agilent Technologies 7500cx series, Santa Clara, CA, USA) was used with sample introduction via a micromist concentric nebuliser (Glass expansion, Port Melbourne, VIC, Australia) and a Scott type double pass spray chamber cooled to 2 °C. The sample solution and the spray chamber waste were carried with the aid of a peristaltic pump (Watson-Marlow, Wetherill Park, NSW, Australia). ICP-MS extraction lens conditions were selected to maximise the sensitivity of a 1% HNO_3_:HCl solution containing 1 ng/ml of lithium, cobalt, yttrium, cerium and thallium. Helium was added into the octopole reaction cell to reduce interferences. Calibration curves were constructed and the results analysed using Masshunter software (version 2014, Agilent Technologies, Santa Clara, CA, USA).

A certified calibration standard containing lithium (Li), beryllium (Be), boron (B), sodium (Na), magnesium (Mg), aluminium (Al), calcium (Ca), vanadium (V), chromium (Cr), manganese (Mn), iron (Fe), cobalt (Co), nickel (Ni), copper (Cu), zinc (Zn), arsenic (As), selenium (Se), strontium (Sr), molybdenum (Mo), silver (Ag), cadmium (Cd), antimony (Sb), barium (Ba), lanthanum (La), europium, (Eu), holmium (Ho), ytterbium (Yb), thallium (Tl), lead (Pb), bismuth (Bi), thorium (Th), uranium (U), phosphorus (P) andmercury (Hg) standard was obtained from Choice Analytical, (Thornleigh, NSW, Australia). Baseline nitric acid (HNO_3_) and hydrogen peroxide (H_2_O_2_) were purchased from Choice Analytical (Thornleigh, NSW, Australia).

### 2.4. Fourier Transform Infrared Spectroscopy

FT-IR was carried out using a Thermo Scientific Nicolet 6700 spectrometer (Waltham, MA, USA).with a DTGS (Deuterated Tri Glycine Sulfate) (Waltham, MA, USA) detector. The powder samples were dispersed in Potassium Bromide discs and all spectra were obtained using 4 cm^−1^ resolution and 64 scans at room temperature.

### 2.5. Protein Extraction from Wallaby Bone

**Figure 1:**
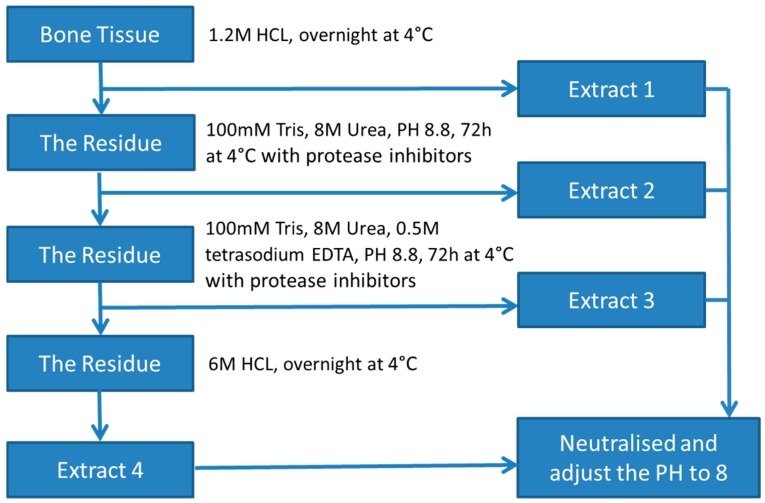
The flow chart of sequential protein extractions method for wallaby bone.

The protein extraction process is summarised in Figure 1. Bone fragments (3 × 3 g) were incubated in 1.2 M HCl (10 mL for each sample) at 4 °C overnight to demineralise bone tissue. The supernatant was collected as Extract 1 (E1) after centrifugation at 4000 rpm for 15 min at 4 °C. The pelleted material was washed with water and extracted for 72 h at 4 °C in a lysis buffer containing 8 M urea, 100 mM Tris-HCl (pH 8.8) and a protease inhibitor cocktail. The supernatant was then collected as Extract 2 (E2) after centrifugation. The pelleted material was extracted further for 72 h at 4 °C in a lysis buffer containing 8 M urea, 100 mM Tris-HCl, 0.5 M tetrasodium EDTA (pH 8.8) and a protease inhibitor cocktail. The supernatant was collected as Extract 3 (E3) after centrifugation. Finally, the remaining pellet was incubated in 6 M HCl overnight at 4 °C. The solution was collected as Extract 4 (E4) after centrifugation [12]. The acidic extracts were neutralised and the pH adjusted to ~8.

### 2.6. Protein Determination with Bradford Assay Kit

Disulfide bonds in the protein samples were reduced using dithiothreitol (20 mM final concentration, Bio-Rad, Hercules, CA, USA). The samples were then incubated at room temperature for 45 min, before alkylation with acrylamide monomers (20 mM final concentration, Bio-Rad), and then incubated at room temperature for 45 min. The protein was precipitated by adding 5 volumes of acetone (1:5, *v*/*v*). After centrifugation, the pellet was resuspended with a minimum volume of 8 M urea, 100 mM NH_4_HCO_3_ using a sonic probe. Protein concentrations in the four extracts of individual wallaby femur, tibia and fibula were determined using the Bradford assay kit (Bio-Rad).

### 2.7. Protein Concentration Assay by SDS-PAGE System

The protein concentrations of E1, E2, E3 and E4 samples were ascertained using SDS-PAGE. Samples diluted to 1:10 and 1:50 and a serial dilution of albumin (1 mg/mL) were mixed with Laemelli sample Buffer (Bio-Rad). Forty microliters of each sample was loaded onto 26-well Criterion™ TGX™ Precast Gels (Bio-Rad), and run at 160 V for 3 min. The gel was fixed and then stained with Flamingo™ Fluorescent stain (Bio-Rad). The density of bands was quantified using Quantity One software (v4.6.1, Bio-Rad) based on the BSA standard curve.

The E4 sample contained salt which was subsequently removed using Bio-spin 6 chromatography columns (Bio-Rad), and then dilutions using ratios of 1:2, 1:4 and 1:8 were carried out on the E4 sample and subjected to SDS = PAGE which showed the salt had been removed.

Approximately 100 µg of protein was diluted with 50 mM NH_4_HCO_3_ to adjust the urea concentration to 1 M. Trypsin (1 µL, MS grade, 1 µg/µL, Sigma-Aldrich, St. Louis, MO, USA) was added to each tube, and the sample tubes were incubated overnight at 37 °C. The digested samples were centrifuged at maximum speed for 5 min prior to solid phase extraction using HLB columns (Silicycle^®^, Quebec, QC, Canada) to recover the peptides. Samples were concentrated to approximately 15 µL (E1), or 25 µL (E2, E3 and E4).

### 2.8. Liquid Chromatography–Mass Spectrometry (LC-MS/MS)

Using an Eksigent AS-1 autosampler connected to a Tempo nanoLC system (Eksigent, Dublin, CA, USA) as previously described [13], 10 µL of each combined extract sample was loaded at 20 µL/min with MS buffer A (2% Acetonitrile + 0.2% Formic Acid) onto a C8 trap column (Michrom Bioresources, Auburn, CA, USA). After washing the trap for three min, the peptides were washed off the trap at 300 nL/min onto a PicoFrit column (75 µm ID × 150 mm; New Objective, Woburn, MA, USA) packed with Magic C18AQ resin (Michrom Bioresources, Auburn, CA, USA), then eluted from the column and into the source of a QSTAR Elite hybrid quadrupole-time-of-flight mass spectrometer (AB Sciex, Eksigent, Dublin, CA, USA) using the following program [14,15]: 5%–30% MS buffer B (98% acetonitrile + 0.2% formic acid) over 60 min, 30%–80% MS buffer B over 3 min, 80% MS buffer B for 2 min, 80%–85% for 3 min. The eluting peptides were ionised at 2300 V. An Intelligent Data Acquisition (IDA) experiment was performed, with a mass range of 350–1500 Da continuously scanned for peptides of charge state 2+–5+ with an intensity of more than 30 counts/s. The selected peptides were fragmented and the product ion fragment masses measured over a mass range of 100–1500 Da. The mass of the precursor peptide was then excluded for 120 s.

### 2.9. MS/MS Data Analysis and Protein Identification

The MS/MS data files produced were searched using Mascot Daemon (version 2.4; Perkins, D.N. 1999, Boston, MA, USA) against the LudwigNR database (comprised of the UniProt, plasmoDB and Ensembl databases, vQ213) with the following parameter settings: Fixed modifications: none; Variable modifications: propionamide, oxidised methionine and deamidated asparagine; Enzyme: semitrypsin; Number of allowed missed cleavages: 3; Peptide mass tolerance: 100 ppm; MS/MS mass tolerance: 0.2 Da; Charge state: 2+, 3+ and 4+. The results of the search were then filtered by including only protein hits with at least one unique peptide (Bold Red) and excluding peptide hits with a p-value greater than 0.05. Peptides identified by the Mascot were further validated using Scaffold (v4.0, Proteome Software, Portland, OR, USA) and by manual inspection of the MS/MS spectra for the peptide to ensure the b- and y-ion series were sufficiently extensive for an accurate identification. Peptide identifications were accepted if they could be established at a probability greater than 95.0% as specified by the Peptide Prophet algorithm [16]. Protein identifications were accepted if they could be established at probability greater than 80.0% as assigned by the Protein Prophet algorithm [17].

### 2.10. In Vitro Evaluation of Wallaby Bone Extracts on Osteogenic Elements

To determine beneficial effect of wallaby bone extracts (WBE) on bone metabolism, osteogenic elements of calcium (Ca) and alkaline phosphatase (ALP) activity were measured in osteogenic cells derived from adipose tissue-derived stem cells (ADSCs) with and without WBE treatment. In brief, WBEs (1, 5 and 10 µg/mL) were added into Dulbecco's Modified Eagle Medium (DMEM) Glutmax/F12 (Gibco^®^, Waltham, MA, USA) medium containing 0.1 μM dexamethasone, 50 µM ascorbate-2-phosphate, 10 mM β-glycerophosphate and 10% foetal bovine serum during 14 days of human ADSCs differentiation into osteogenic cells, as previously described [18], and culture medium treatment as a control. At the end of the experiment, WBEs treated cells with the control cells were harvested for determination of amount of calcium and ALP activity. In brief, cell samples were completely lysed by sonication and the supernatants were collected for measuring amount of calcium and the ALP activity using the automatic biochemistry instrument (ARCHITECT, Abbot Park, IL, USA) according to manufacturer’s instruction.

### 2.11. Statistical Analysis

Quantitative data is expressed as the mean ± SEM. Comparisons across the four groups were done using one-way ANOVA, followed by a post hoc analysis of Tukey’s test to determine significant differences between the two groups, using Prism version 4 (Graph Pad Inc., San Diego, CA, USA). In this process, a *p*-value < 0.05 was considered statistically significant.

## 3. Results

### 3.1. Mineral Analysis

All mineral analyses were conducted in triplicate. The results of the major component analysis are shown in Table 1. The concentrations of moisture, calcium and phosphorus from wallaby bone are 41.24%, 26.21% and 14.72%, respectively.

There is a wide variety of mineral elements at moderate levels in wallaby bone (Table 2), the magnesium content is 870 mg/kg, which is good for magnesium supplementation, and other mineral elements assayed include aluminium, iron, strontium, zinc, barium at 264.44 mg/kg, 151.59 mg/kg, 559.19 mg/kg, 76.94 mg/kg, 88.91 mg/kg, respectively, the contents of which are high and rich.

According to the European Medicines Agency standards, and the Committee for Medicinal Products for Human Use (CHMP) [19], all of the heavy metal concentrations from kangaroo bone are below the human prescribed standards (Table 3). Of importance, the level of arsenic was below the detection limit [20]. However, not all heavy metals have negative effects. For example, copper, iron, zinc, manganese and other elements are essential trace elements to human health because they are not only the main ingredient of anti-free radical substances in the human body, and have anti-ageing effects [21], but also co-factors in various important metalloenzymes and other metalloproteins, such as hemeproteins (haemoglobin, cytochromes etc.) [22,23].

A recent medical study has shown that copper deficiency can lead to bone mineralization disorders and cause senile osteoporosis. Furthermore, it found that iron is an important component of red blood cells that plays an essential role in haematopoiesis. The use of iron supplementation from wallaby bones may prevent iron deficiency anaemia [24].

Manganese and zinc not only play an active role in normal growth, but are also very important to other biological activities; manganese is involved in haematopoiesis, oxidation, reduction, calcium and phosphorus metabolism, bone formation, and the growth and development of body function [25]. Zinc is also a key factor for a healthy immune system.

Therefore, the development of a wallaby bone supplement could offer a simple and effective alternative to calcium and magnesium supplementation, and address damage to the human body caused by a lack of these minerals.

### 3.2. Organic Compounds Analysis

**Figure 2:**
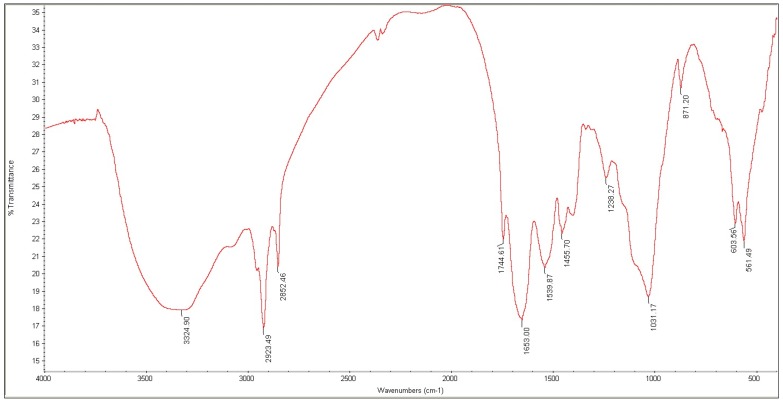
The FTIR spectrum of wallaby bone shows bands originating from protein (mostly amide C=O groups: these include the amide I band at about 1653 cm^−1^, the amide II band at about 1540 cm^−1^ and the amide III band about 1238 cm^−1^) and lipids (e.g., C–H vibrations at about 2923, 2852 and 1744 cm^−1^).

FT-IR spectroscopy was used to measure bulk biochemical characteristics of the meat-and bone-based samples and also provided a check on contamination of the products by organic compounds. The FT-IR spectrum of wallaby bone is shown in Figure 2.

The bands from the mineral component (mostly carbonate and phosphate apatite groups) are those representing phosphate groups at about 1100–950 and 600–550 cm^−1^ (phosphate ν_1_, ν_3_ and ν_4_ respectively) and carbonate groups at about 875 and 1500–1400 cm^−1^ (the ν_2_ and ν_3_ vibrational modes respectively). These spectra are consistent with those of normal bone samples.

### 3.3. Total Protein Content from Four Protein Extracts in Different Bone Samples

To determine the different protein concentrations from the four different extracts from bone samples, 40 extraction samples were collected together and tested using the Bradford assay kit (Bio-rad Laboratories, Hercules, CA, USA) at the same time as the first protein determination. Total protein content of four protein extracts from different types of bone samples was shown in Table 4.

### 3.4. Identification of Peptides and Proteins by Proteome Analysis

**Figure 3:**
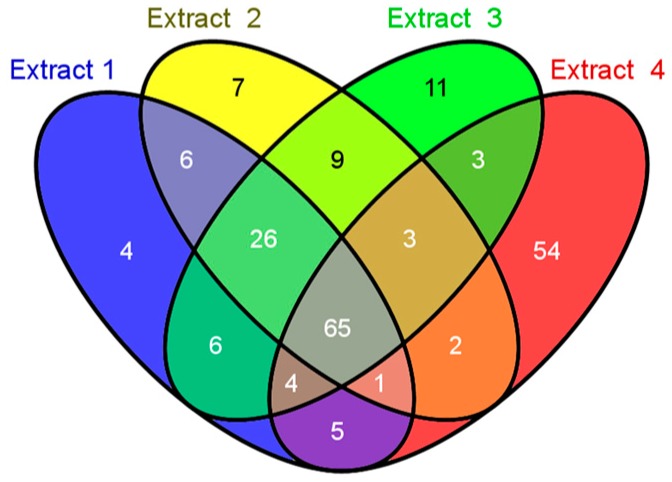
Venn-diagram showing the number of unique proteins that overlap in the four wallaby bone protein extracts, as listed from Table 5.

According to the volume loaded into the LC/MS/MS instrument, the quantity of peptide present was 5 µg for each extract. The filtering criterion was set at 95.0% minimum Peptide Thresholds and the false-positive prophet rate was 0.58%. For the uniquely identified proteins, the filtering criterion was 50.0% minimum 1 min peptides and the false-discovery rate was 2.4% and the peptides and proteins were identified with high confidence. The number of unique peptides and unique proteins from the four extractions are listed in Table 2. E4 contained 481, 417 and 137 unique spectra, peptides and proteins, respectively, as seen in Table 5 and Figure 3, which was the highest amount among the extractions. However, previous studies have suggested that prolonging the instrument acquisition time and the loading volume may have produced different results [12,15].

### 3.5. Biological Process Classification of the Identified Proteins

**Figure 4:**
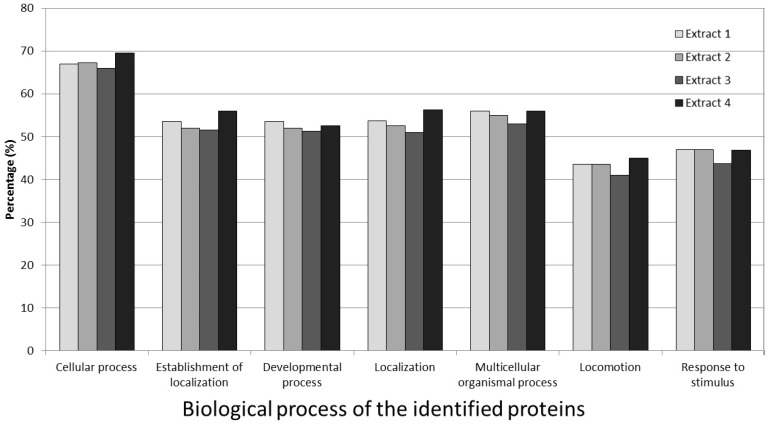
Percentage classification into biological process categories for proteins from the four extraction groups, using Gene Ontology Terms. All results were from the Intelligent Data Acquisition analysis and acquired by manual inspection with Scaffold software.

A total of 171 identified proteins (83.0%) were classified into biological process categories. These classifications were based on common gene ontology terms and can be seen in Figure 4. The bone remodelling proteins found within these categories include osteomodulin, osteopontin, osteoglycin, osteocalcin, bone sialoprotein 2 and decorin.

### 3.6. Cellular Component of the Identified Proteins

**Figure 5:**
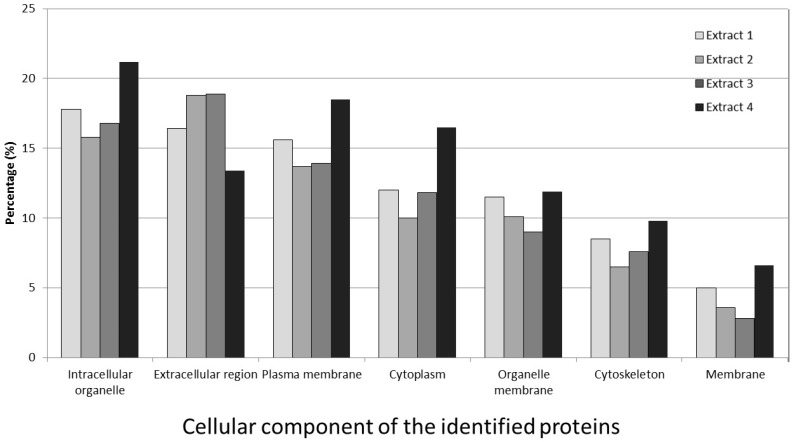
Percentage classification into cellular components for proteins from the four extraction groups, using Gene Ontology Terms. All results were from the Intelligent Data Acquisition analysis and acquired by manual inspection with Scaffold software.

A total of 60 identified proteins (29.1%) were classified as cellular components, the cellular components from individual sample extractions are classified as shown in Figure 5. The highest proportion of identified proteins was found in the intracellular organelle, amounting to 16%–21%. In descending order, the proteins were found in the extracellular region (13.5%–18.5%), the organelle (13.5%–18.5%), the cytoplasm (10%–16.5%) and the cytoskeleton (6.5%–9.8%).

### 3.7. Protein Role Assignment of the Identified Proteins

**Figure 6:**
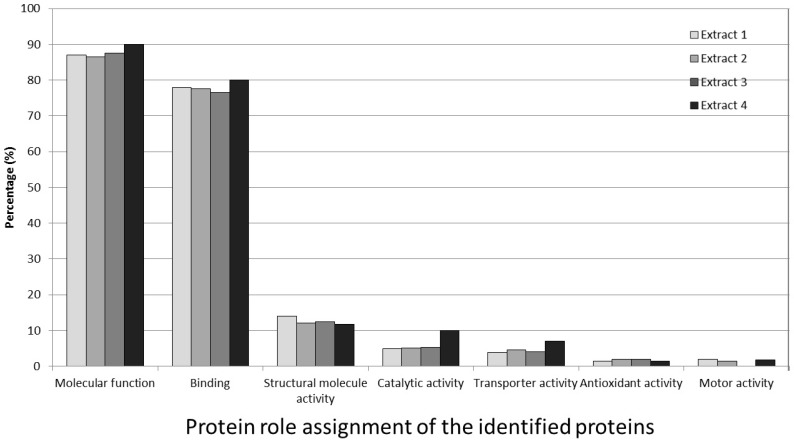
Percentage classification according to molecular function for proteins from the four extraction groups, using Gene Ontology Terms. All results were from the Intelligent Data Acquisition analysis and acquired by manual inspection with Scaffold software.

A total of 178 identified proteins (86.4%) were classified according to protein function. The classification of identified proteins from the individual sample extractions according to molecular function is shown in Figure 6. This graph shows the identified proteins based on their known physiological functions. The interesting thing found here was that there were only two main groups were detected; binding group (77%–80%) and molecular function group (87%–90%) as shown in Figure 6. The only other minor group detected was structural molecule activity group (12%–14%) with the rest of the groups were lower than 10%, as seen in Figure 6.

The molecular function group had the largest number of proteins identified. This broad category contained many proteases and enzymes involved in the stimulation and proliferation of bone cells. This important discovery will be the main focus of our further study. The second largest number of proteins was present in the binding function group.

### 3.8. Calcium Concentration and Alkaline Phosphate Activity in WBE Treated Osteogenic Cells

**Figure 7:**
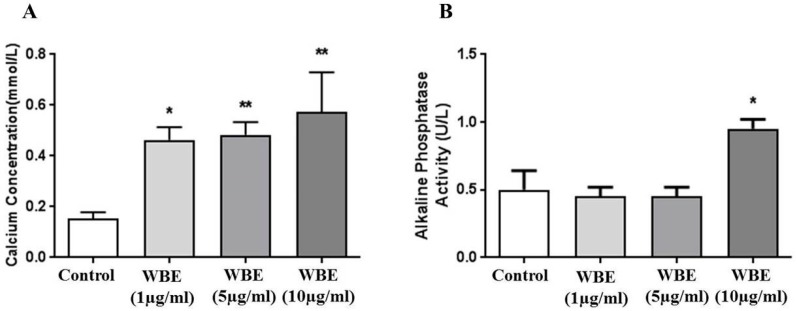
Calcium content (**A**) and ALP activity (**B**) in osteogenic cells derived from adipose tissue-derived stem cells (ADSCs) with wallaby bone extracts (WBE) treatments (1, 5 and 10 µg/mL). Data from three separate experiments, * *p* < 0.05 and ** *p* < 0.01 vs. control.

The calcium concentrations were significantly higher by 3.0-, 3.1- and 3.7-fold, respectively, in WBE treated cells compared to the control cells (Figure 7A) but only high dose of WBE treatment (10 µg/mL) enhanced ALP activity (Figure 7B).

## 4. Discussion

From the chemical analysis of wallaby bone, the minerals analysis showed that there was not a significant difference between the femur, fibula and tibia samples (Table 3). When these results are compared with other domestic animals’ bone, the concentrations of calcium and phosphorus in wallaby bone are significantly higher than in alpaca bone, beef bone, pork bone, lamb bone and chicken bone (Table 6) previously reported [21,26]. The most interesting finding is the 2:1 ratio of calcium and phosphorus in wallaby bone, which is similar to the calcium to phosphorus ratio in human bone. This ratio may make the substance of wallaby bone readily absorbed for consumption. In addition, wallaby bone contains high levels of other essential minerals with heavy metal concentrations below the human prescribed standard.

Protein analysis has revealed that the femur contains the highest amount of protein. Extract 4 from each type of bone had the highest amount of protein among the four extracts. This result matched the protein identification results from the LC/MS/MS. A total number of 2038 spectra, 206 unique proteins and 137 clusters were identified. The analysis of Extract 4 also presented the largest number of spectra, peptides and proteins. When the proteins from the extracts were classified according to cellular component, biological process and protein role assignment, the largest component of the four extractions was also Extract 4 among groups; this was consistent with the protein assay.

These results may be attributed to a high abundance of proteins or protein fragments existing in this fraction (Extract 4) that may suppress the identification of other proteins [12]. Additionally, among those 206 unique proteins identified, up to 65 (31.6%) were found in all four extracts, while 4 (1.9%) proteins in Extract 1, 7 (3.3%) proteins in Extract 2, 11 (5.3%) proteins in Extract 3, and 54 (26.2%) proteins in Extract 4 were uniquely observed (Figure 3). Even the amount of overlap between proteins that had been identified was quite high in the fractions from different extraction steps. The different extraction methods (Extracts 1 to 4) still have a lot of unique proteins, particularly Extract 4.

The most interesting results found in the gene ontology and cell function groups of those identified proteins were that the major components included proteases and enzymes involved in the stimulation and proliferation of bone cells. Normally, the non-collagenous proteins only make up 10% of total bone protein content, while a majority of the biologically important proteins are naturally relatively low. However, in the proteome analysis study, we found that the cellular process and organismal process proteins of biological process classification were identified up to 69% and 54%, respectively, indicating that high amounts of bone remodelling proteins, like osteomodulin, osteopontin, osteoglycin, osteocalcin, bone sialoprotein-2 and decorin may exist in wallaby bone.

This study was designed mainly to characterize bone beneficial components from Australian Wallaby bone but the in vitro evaluation of wallaby bone extracts in the differentiation of ADSCs into osteogenic cells showed the WBEs increased deposition of calcium in osteoblasts with enhanced ALP activity at a high dose. In increased ALP activity and total calcium concentration by the nutrients have demonstrated the positive effects on the homeostasis of bone [27]. Although more investigation is required, these data highlights a view of the future study on WBE as supplements to prevent or treat bone disorder, such as osteoporosis and bone fracture.

Overall, the results of mineral and protein analysis suggest that substance from Australian wallaby bone may have potential to be beneficial for mineral deficiency related to osteoporosis. Future studies using an animal model of osteoporosis to investigate the therapeutic effect of wallaby bone extract on bone formation, differentiation and proliferation is warranted.

## Figures and Tables

**Table 1:** Quantity of major components in wallaby bones.

Bone Samples	Water (%)	Ca (%)	*p* (%)
Femur	54.44 ± 4.09	22.72 ± 8.14	12.36 ± 4.46
Fibula	34.05 ± 5.90	27.54 ± 1.46	16.47 ± 6.24
Tibia	35.23 ± 6.34	27.33 ± 3.16	14.92 ± 1.16
Average	41.24 ± 11.45	26.21 ± 6.87	14.72 ± 4.64

Results show different bone parts (femur, fibula and tibia) derived from three individual wallaby samples (*n* = 3).

**Table 2:** Quantity of medium level minerals in wallaby bones.

Bone Samples	Na	Mg	Al	Fe	Sr	Ba
	(mg/kg)	(mg/kg)	(mg/kg)	(mg/kg)	(mg/kg)	(mg/kg)
Femur	5.48 × 10^4^ ± 0.53	0.99 × 10^3^ ± 0.03	238.77 ± 22.99	135.04 ± 3.08	495.00 ± 15.71	89.16 ± 13.54
Fibula	5.16 × 10^4^ ± 0.24	0.78 × 10^3^ ± 0.14	292.71 ± 13.37	166.64 ± 1.38	611.83 ± 23.96	92.96 ± 14.00
Tibia	4.40 × 10^4^ ± 0.81	0.83 × 10^3^ ± 0.05	261.84 ± 14.85	153.08 ± 4.00	570.75 ± 11.59	84.62 ± 9.10
Average	5.01 × 10^4^ ± 1.55	0.87 × 10^3^ ± 0.21	264.44 ± 27.06	151.59 ± 15.85	559.19 ± 59.26	88.91 ± 4.18

Results show different bone parts (femur, fibula and tibia) derived from three individual wallaby samples (*n* = 3).

**Table 3:** Quantity of heavy metals and micro minerals in wallaby bones.

Bone Samples	Pb	Ni	Mn	Cu	Cr	Se	Zn
	(mg/kg)	(mg/kg)	(mg/kg)	(mg/kg)	(mg/kg)	(mg/kg)	(mg/kg)
Femur	0.15 ± 0.01	3.97 ± 0.86	1.31 ± 0.21	1.11 ± 0.33	1.08 ± 0.19	0.71 ± 0.03	71.65 ± 5.52
Fibula	0.41 ± 0.01	1.36 ± 0.45	2.82 ± 0.36	1.84 ± 1.13	1.30 ± 0.25	0.76 ± 0.04	81.74 ± 6.46
Tibia	0.16 ± 0.08	0.05 ± 0.02	1.31 ± 0.24	1.12 ± 0.90	0.09 ± 0.05	0.69 ± 0.05	77.44 ± 8.27
Average	0.24 ± 0.15	1.79 ± 1.99	2.11 ± 0.76	1.36 ± 0.42	0.82 ± 0.64	0.72 ± 0.12	76.94 ± 5.06

Results show different bone parts (femur, fibula and tibia) derived from three individual wallaby samples (*n* = 3).

**Table 4:** Total protein content of protein extracts (mg/g of bone) from different types of bone.

Bone Protein Extracts	Femur (mg/g)	Tibia (mg/g)	Fibula (mg/g)
Extract 1	0.709 ± 0.07	0.611 ± 0.08	0.236± 0.03
Extract 2	0.922 ± 0.04	0.718 ± 0.04	0.305 ± 0.02
Extract 3	0.152 ± 0.02	0.092 ± 0.02	0.008 ± 0.05
Extract 4	0.70 ± 0.04	0.787 ± 0.05	0.613 ± 0.01
Total	2.48 ± 0.1.36	2.21 ± 0.11	1.23 ± 0.13

**Table 5:** Quantity of protein extracted, number of peptides and proteins identified.

Wallaby Bone Protein Extracts	Number of Unique Spectra	Number of Unique Peptides	Number of Unique Proteins	Number of Clusters
Extract 1	334	293	117	74
Extract 2	362	311	119	72
Extract 3	409	362	127	79
Extract 4	481	417	137	85

The quantity of protein extracted, number of peptides and proteins identified from the four protein extracts were identified using the sequential extraction protocol by auto-searching with Scaffold software.

**Table 6:** Average of major component in wallaby bones compared with the bones of domestic animals.

Bone Samples	Water (%)	Ca (%)	*p* (%)
Wallaby bone	41.24	26.21	14.72
Alpaca Bone	41	20	8.8
Beef Bone	64.2	5.1	2
Pork Bone	66.7	4.5	2.2
Lamb Bone	65.1	3.4	1.6
Chicken Bone	65.6	1	0.5
